# Evolutionary Relationship and Structural Characterization of the *EPF/EPFL* Gene Family

**DOI:** 10.1371/journal.pone.0065183

**Published:** 2013-06-03

**Authors:** Naoki Takata, Kiyonobu Yokota, Shinya Ohki, Masashi Mori, Toru Taniguchi, Manabu Kurita

**Affiliations:** 1 Forest Bio-Research Center, Forestry and Forest Products Research Institute, Hitachi, Japan; 2 Kanazawa University Graduate School of Medical Sciences, Kanazawa, Japan; 3 Center for Nano Materials and Technology, Japan Advanced Institute of Science and Technology, Nomi, Japan; 4 Research Institute for Bioresources and Biotechnology, Ishikawa Prefectural University, Nonoichi, Japan; 5 Forest Tree Breeding Center, Forestry and Forest Products Research Institute, Hitachi, Japan; University of Nottingham, United Kingdom

## Abstract

EPF1-EPF2 and EPFL9/Stomagen act antagonistically in regulating leaf stomatal density. The aim of this study was to elucidate the evolutionary functional divergence of *EPF/EPFL* family genes. Phylogenetic analyses showed that *AtEPFL9*/*Stomagen*-like genes are conserved only in vascular plants and are closely related to *AtEPF1*/*EPF2*-like genes. Modeling showed that EPF/EPFL peptides share a common 3D structure that is constituted of a scaffold and loop. Molecular dynamics simulation suggested that AtEPF1/EPF2-like peptides form an additional disulfide bond in their loop regions and show greater flexibility in these regions than AtEPFL9/Stomagen-like peptides. This study uncovered the evolutionary relationship and the conformational divergence of proteins encoded by the *EPF/EPFL* family genes.

## Introduction

Stomata are pores on the aerial surfaces of land plants that mediate gas exchange between plants and their environment. Stomata are found in mosses and hornworts in bryophytes and in vascular plants including lycophytes, ferns, gymnosperms and angiosperms [1]. In the evolution of land plants, stomatal density significantly shifted in response to fluctuations in the atmospheric environment such as CO_2_ concentration. Ancient land plants from the Silurian to the Devonian had a low stomatal density relative to extant land plants [2]. By the late Devonian/early Carboniferous, the density had increased by up to 100 times in response to a dramatic decline in the atmospheric CO_2_ concentration [3], which resulted in morphological advances such as planate leaves in vascular plants [4]. This evolutionary innovation in leaf tissues implies that early vascular plants had developed molecular systems elevating their stomatal density when they perceived the atmospheric fluctuation.

The *EPF*/*EPFL* (*epidermal patterning factor*/*EPF-like*) gene family encodes plant-specific secretory peptides, several of which play a role in controlling stomatal density and patterning in the plant epidermis [5]. Mature EPF/EPFL peptides have conserved six or eight cysteine residues that form intramolecular disulfide bonds [6]. The structure of *Arabidopsis thaliana* (At) EPFL9/Stomagen inferred by NMR indicates that the peptide is composed of two structural parts, a scaffold and a loop. The scaffold consists of a pair of ß-strands forming an anti-parallel ß-sheet supported by three disulfide bonds with a one-turn 3_10_-helix, which is structurally required for the activity of the peptides. The loop domain connecting the two ß-strands contributes to the functional specificity of each EPF/EPFL peptide during stomatal development. In the model plant *A. thaliana*, AtEPF1 and AtEPF2 have a negative role in leaf stomatal development [7]–[9], in which an additional disulfide bond is formed between two cysteine residues in the loop region. Intriguingly, AtEPFL9/Stomagen lacking an additional disulfide bond acts as a positive regulator of leaf stomatal density and has an antagonistic action on AtEPF1 and AtEPF2 [6], [10].

The genes regulating stomatal density and patterning are well conserved from basal land plants. The small peptides AtEPF1 and AtEPF2 form ligand-receptor complexes with TOO MANY MOUTHS (TMM) and ERECTA family members in the *A. thaliana* [11]. TMM and the ERECTA family play roles as putative receptors for EPF peptides to initiate a signal cascade for stomatal development. The basal land plant *Physcomitrella patens*, which develops stomata around the base of its sporophytes [12], retains one copy of the *EPF/EPFL* gene, one copy of *TMM* and six copies of *ERECTA* family genes [13]–[15]. Intriguingly, an EPF/EPFL ortholog in *P. patens* has more similarity to negative regulators AtEPF1 and AtEPF2 than to positive regulator AtEPFL9/Stomagen, whereas in the genome of the lycophyte *Selaginella moellendorffii*, both *AtEPFL9*/*Stomagen*-like genes and *AtEPF1/EPF2*-like genes are conserved [15]. However, the evolutionary relationships between the negative and positive regulators are still unclear in the *EPF*/*EPFL* gene family.

In the present study, we examined the phylogenetic relationship of and structurally characterized the *EPF*/*EPFL* gene family to clarify evolutionary functional divergence between the negative and positive regulators. To this end, our extensive analysis reconstructed phylogenetic trees using *EPF*/*EPFL* genes in land plants and predicted the 3D structures of their products. The 3D structures were subjected to a computer simulation to analyze their molecular dynamical properties. This study revealed that the *AtEPFL9*/*Stomagen*-like genes conserved in vascular plants are closely related to *AtEPF1/EPF2*-like genes and that AtEPF1/EPF2-like peptides have greater flexibility in the loop region than AtEPFL9/Stomagen-like peptides. Our data allowed us to explore the evolutionary history of leaf stomatal density in early land plants.

## Materials and Methods

### Phylogenetic Analysis

*EPF/EPFL* genes were retrieved from genomic database for *Arabidopsis thaliana* (The Arabidopsis Information Resource, TAIR). To identify *EPF/EPFL* genes in *Carica papaya* (Cp), *Medicago truncatula* (Mt), *Oryza sativa* (Os), *Picea glauca* (Pg), *Physcomitrella patens* (Pp), *Populus trichocarpa* (Pt), *Selaginella moellendorffii* (Sm) and *Sorghum bicolor* (Sb), TBLASTN searches were performed against the genomic databases using amino acid sequences encoded by *EPF/EPFL* genes of *A. thaliana* (At) as queries: Rice Annotation Project Database for *O. sativa*, The Gene Index Project for *P. glauca*, and Phytozome v8.0 [16] for *C. papaya*, *M. truncatula*, *P. trichocarpa*, *P. patens*, *S. bicolor* and *S. moellendorffii*. Genes that retained the typical ß-sheet scaffold and the loop domain were retrieved from these genomic databases. Accession numbers or locus IDs of *EPF/EPFL*s are described in Table S1. For predicted genes lacking a conserved portion of the *EPF/EPFL* gene, the open reading frame of the gene was repredicted using an assembled EST sequence made by the PASA tool [17] in the Phytozome database. For unpredicted genes in the *S. moellendorffii* genomic database, the protein-coding region was predicted by the Fgenesh+ program [18]. Amino acid sequences for the C-terminal mature peptide region were aligned using the ClustalW program [19]. The number of amino acid substitutions between each pair of EPF/EPFL proteins was estimated by the Jones-Taylor-Thornton (JTT) model [20] with the complete-deletion option. From estimated numbers of amino acid substitutions, phylogenetic trees were reconstructed by the minimum evolution (ME) and neighbor-joining (NJ) methods. Bootstrap values were calculated with 1,000 replications. These procedures were performed using MEGA5 software [21].

### Modeling

Three-dimensional structures of EPF/EPFL peptides were modeled with Modeller version 9.9 software [22]. The structure of AtEPFL9/Stomagen determined by NMR was employed as the template and SS-bonds were allowed to form during modeling. Structural figures were prepared using the MOLMOL graphics program [23].

### Molecular Dynamics Simulation

The modeling structures were used as the starting structure for molecular dynamics (MD) simulation. The calculation was performed with the GROMACS program [24] using an AMBER99-SB force field [25]. Each structure was placed in the center of a 60 Å × 60 Å × 60 Å cubic box with periodic boundary conditions and solvated by SPC/E water molecules [26]. Na^+^ or Cl^-^ counterions were added to satisfy the electroneutrality condition for each system. Berendsen temperature coupling and Parrinello-Rahman pressure coupling were used to keep the system in a stable environment (300 K, 1 bar), and the coupling constants were set to 0.1 ps. The particle mesh Ewald (PME) algorithm [27] was employed to calculate long-range electrostatic interactions with a cutoff value of 1.0 nm, and a cutoff of 1.0 nm was set for van der Waals interactions. The LINCS algorithm [28] for bond constraints was applied. Each system was energy-minimized with a steepest-descent algorithm for 1,000 steps; then the solvent, ions were equilibrated for 1 ns in NTP and NVT ensembles, respectively. Finally, all constraints were removed and a 10 ns MD simulation was performed for each system. All the trajectories were stored every 200 ps for further analysis.

Trajectories for each structure in aqueous solution were analyzed to obtain structural and dynamic properties using the GROMACS analysis tools package, including the root mean square fluctuation of the residues (RMSF), root mean square deviation (RMSD), and secondary structure prediction, which were performed using the STRIDE program [29].

## Results

### Phylogenetic Analysis of *EPF*/*EPFL* Gene Family

**Figure 1:**
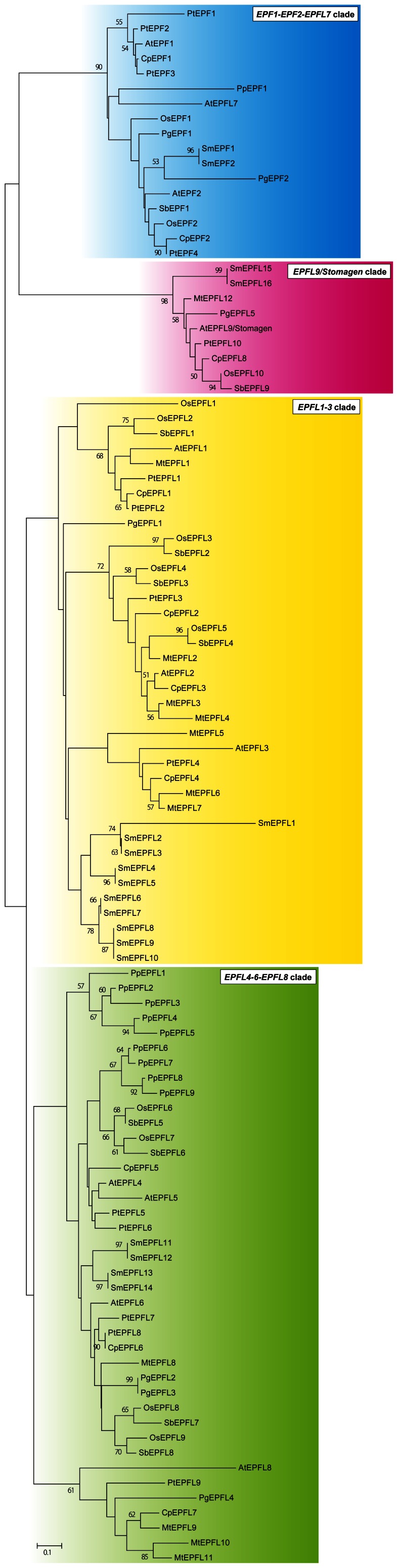
Phylogenetic tree of *EPF/EPFL* genes. Amino acid sequences for the mature peptide region were aligned using the ClustalW program. The phylogenetic tree was reconstructed by the ME method from the numbers of amino acid substitutions estimated by the JTT model. The numerals at the branch indicate bootstrap values calculated by the ME method with 1,000 replications. Bootstrap values >50% are shown.

*EPF/EPFL* genes are conserved in land plants but not in algae such as *Chlamydomonas*, *Ostreococcus* and *Cyanidioschyzon*. Phylogenetic trees clearly showed that *EPF/EPFL* genes separated into four clades (*EPF1-EPF2-EPFL7* clade, *EPFL9/Stomagen* clade, *EPFL1*-*3* clade and *EPFL4*-*6-EPFL8* clade; the clades were named after respective *EPF/EPFL*s in *A. thaliana*.) in land plants (Fig. 1, Fig. S1, Dataset S1). The *EPF1-EPF2-EPFL7* clade and the *EPFL4*-*6-EPFL8* clade contained genes from bryophyte and vascular plants including lycophytes, gymnosperms, monocots and eudicots. On the other hand, the *EPFL9/Stomagen* clade and the *EPFL1*-*3* clade were constituted of genes only from vascular plants. The negative regulators such as AtEPF1 and AtEPF2 were found in the *EPF1-EPF2-EPFL7* clade. AtEPFL9/Stomagen, the antagonist of AtEPF1 and AtEPF2, was a member of the *EPFL9/Stomagen* clade. The phylogenetic trees showed close relationships between the *EPF1-EPF2-EPFL7* clade and the *EPFL9/Stomagen* clade and between the *EPFL1*-*3* clade and the *EPFL4*-*6-EPFL8* clade in the *EPF/EPFL* gene family.

### Three-dimensional Structural Models of EPF/EPFL Peptides

**Figure 2:**
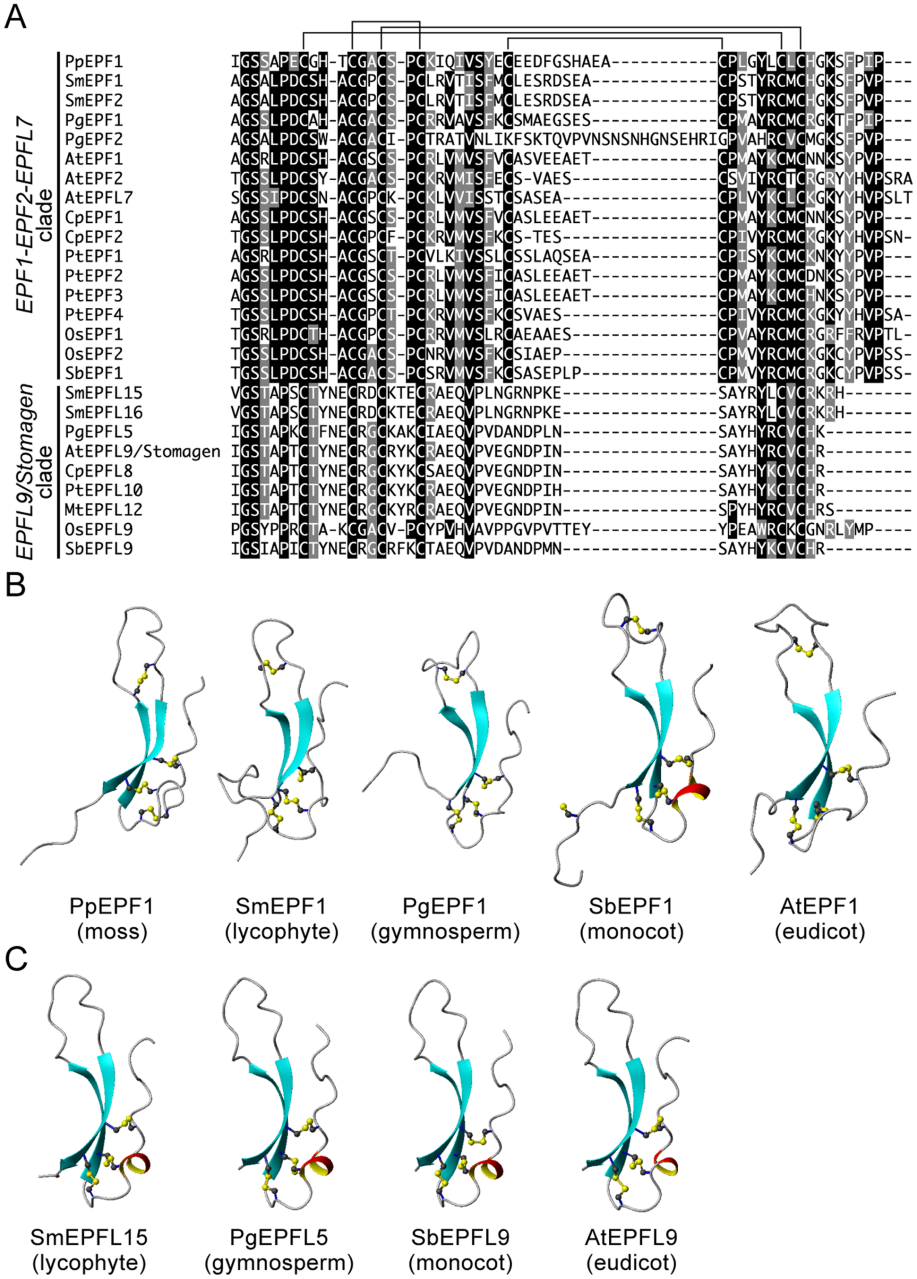
Structures of AtEPF1/EPF2-like peptides and AtEPFL9/Stomagen-like peptides. (A) Primary structures of AtEPF1/EPF2-like peptides and AtEPFL9/Stomagen-like peptides in land plants. Sequence alignment was generated by the ClustalW program. Pairs of cysteine residues forming disulfide bonds predicted for *A. thaliana* EPF/EPFL genes are indicated by lines. (B) and (C) Ribbon models of EPF/EPFL peptides. Structural models shown here were generated by homology modeling. The structure of AtEPFL9/Stomagen determined by NMR was used as the template for the homology modeling. A model of the disulfide bonds is shown as a ball-and-stick representation.

The phylogenetic analysis showed that the negative regulators AtEPF1 and AtEPF2 were closely related to the antagonist AtEPFL9/Stomagen. To characterize peptide sequences in the *EPF1-EPF2-EPFL7* clade and the *EPFL9/Stomagen* clade, we analyzed their 1D structures. The prepropeptides for AtEPF1, AtEPF2 and AtEPFL9/Stomagen contain a signal peptide at their N-terminal regions [15]. A putative signal peptide was identified in genes from mosses, lycophytes, gymnosperms, monocots and eudicots using SignalP software [30] (Table S2). In the C-terminal mature peptide regions, six-cysteine residues located in a scaffold region were well conserved for all genes in both clades (Fig. 2A). Additional two-cysteine residues in the loop region were only conserved within the peptides in the *EPF1-EPF2-EPFL7* clade except for PgEPF2.

We next translated their 1D sequence information into 3D structural features. Homology modeling was performed to examine structural properties of the peptides in the *EPF1-EPF2-EPFL7* clade and the *EPFL9/Stomagen* clade. Fig. 2B and 2C show 3D structures of the representative peptides from a moss (*P. patens*), a lycophyte (*S. moellendorffii*), a gymnosperm (*P. glauca*), a monocot (*S. bicolor*) and a eudicot (*A. thaliana*) in evolutionary lineages of plants. The 3D structure modeling clearly showed that the peptides shared an AtEPFL9/Stomagen-like structure that is composed of a consensus scaffold and a long loop. In the scaffold regions, three disulfide bonds were formed between an anti-parallel ß-strand and a one-turn 3_10_-helix in SbEPF1, AtEPF1, SmEPFL15, PgEPFL5, SbEPFL9 and AtEPFL9. In PpEPF1, SmEPF1 and PgEPF1 peptides, one or two disulfide bonds were adopted between adjacent amino acids of the first ß-strand and the helix. The AtEPF1/EPF2-like peptides form an additional disulfide bond in their loop regions, although the AtEPFL9/Stomagen-like peptides do not. The PgEPF2, lacking any additional two-cysteine residues, does not retain a disulfide bond in its loop even though it is a member of the *EPF1-EPF2-EPFL7* clade (Fig. S2A).

### MD Simulation of EPF/EPFL Peptides

**Figure 3:**
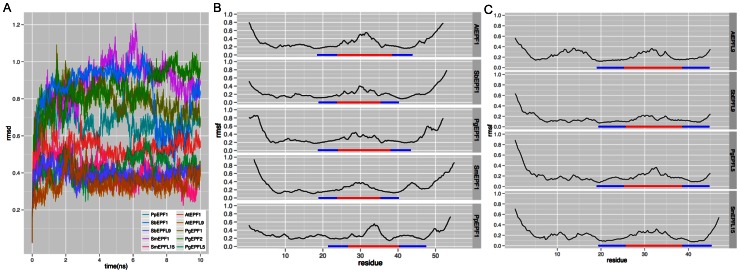
Results of MD simulation of AtEPF1/EPF2-like peptides and AtEPFL9/Stomagen-like peptides. (A) RMSD values (in nm) during a 10 ns molecular dynamic simulation. (B) and (C) RMSF value (in nm) through the amino acid sequence of each peptide. RMSD indicates conformational changes from the initial structure and RMSF indicates fluctuating area in the molecule. Blue and red bars indicate the ß-strand and loop, respectively.

To clarify conformational divergence between AtEPF1/EPF2-like peptides and AtEPFL9/Stomagen-like peptides, 3D structural stability and flexibility of the EPF/EPFLs were examined by MD simulation. The 10 ns MD simulation was performed for six and four peptides, respectively, in the *EPF1-EPF2-EPFL7* clade and *EPFL9/Stomagen* clade. The RMSD value of the AtEPF1 was higher than that of AtEPFL9/Stomagen (Fig. 3A). Likewise, the values for the AtEPF1/EPF2-like peptides were distinguishable from the AtEPFL9/Stomagen-like peptides. This indicates that the EPF/EPFLs in the *EPF1-EPF2-EPFL7* clade are less conformationally stable than those in the *EPFL9/Stomagen* clade. For the RMSF values plotted through amino acid sequence, AtEPF1 showed a higher value in the loop region than AtEPFL9/Stomagen (Fig. 3B and 3C). Similarly to the *A. thaliana* EPF/EPFLs, the AtEPF1/EPF2-like peptides including moss EPF1 fluctuated more in the loop region than the AtEPFL9/Stomagen-like peptides (Fig. 3B and 3C, Fig. S2B). Taken together, these data suggest that the EPF/EPFLs in the *EPF1-EPF2-EPFL7* clade have greater flexibility in their functional loop regions than those in the *EPFL9/Stomagen* clade.

## Discussion

AtEPF1 and AtEPF2 have an antagonistic role with AtEPFL9/Stomagen in regulating stomatal density and patterning in leaf tissue. Although *EPF/EPFL* genes are widely conserved in land plants, their phylogenetic relationship remains to be determined. Here, we elucidated the evolutionary relationship of *EPF/EPFL* genes in land plants and examined their conformational divergence to deduce their emergence and acquisition of antagonistic function during land plant evolution.

In the phylogenetic trees, *AtEPF1/EPF2*-like genes and *AtEPFL9/Stomagen*-like genes showed a close relationship in *EPF/EPFL* family genes (Fig. 1, Fig. S1). *AtEPF1/EPF2*-like genes constitute the *EPF1-EPF2-EPFL7* clade that contains *AtEPF1* and *AtEPF2*, which negatively regulate stomatal development. *AtEPFL9/Stomagen*-like genes comprise the *EPFL9/Stomagen* clade that includes the positive regulator *AtEPFL9/Stomagen*. Although the *EPF1-EPF2-EPFL7* clade is composed of genes from both bryophytes and vascular plants, the *EPFL9/Stomagen* clade does not conserve any gene from mosses. Collectively, these findings suggest that the *EPF1-EPF2-EPFL7* clade and the *EPFL9/Stomagen* clade may share a common ancestral gene and the latter clade formed after the speciation of bryophytes and vascular plants.

AtEPF1/EPF2-like peptides and AtEPFL9/Stomagen-like peptides display different plasticity in their 3D structures, although they share a common conformation composed of a consensus scaffold and a loop (Figs. 2 and 3). In stomatal development of the model plant *A. thaliana*, both the ß-sheet scaffold and the loop domain are required for activity [6]. Moreover, the loop domain confers the functional specificity of EPF/EPFL peptides. The 3D structure modeling revealed differences in the loop region between AtEPF1/EPF2-like peptides and AtEPFL9/Stomagen-like peptides (Fig. 2). AtEPF1/EPF2-like peptides including the moss EPF1 form a disulfide bond in the loop region, whereas AtEPFL9/Stomagen-like peptides do not. Fig. 3 shows that the functional loop of the AtEPF1/EPF2-like peptides is more flexible than that of the AtEPFL9/Stomagen-like peptides. The additional disulfide bond is not always essential for loop flexibility because PgEPF2 exhibited a higher RMSF value in its loop (Fig. S2B). Amino acid composition and length of loops affect conformational stability, for example, longer inter-domain linkers show the greater flexibility [31]. The difference in the loop flexibility between AtEPF1/EPF2-like peptides and the AtEPFL9/Stomagen-like peptides would be defined by their loop length and additional disulfide bond that restricts the conformational degree of freedom in the loop domain [32]. The flexibility of the functional loop may contribute to the antagonistic function between the AtEPF1/EPF2-like peptides and the AtEPFL9/Stomagen-like peptides in stomatal development.

Our evolutionary and structural analyses revealed that the moss *P. patens* and vascular plants conserve the *AtEPF1/EPF2*-like gene in a structurally similar form to that of negative regulators and that vascular plants including lycophytes retain the *AtEPFL9/Stomagen*-like gene (Figs. 1, 2 and 3). AtEPF1 and AtEPF2 form ligand-receptor modules with TMM and ERECTA family members to initiate a signaling pathway in stomatal development [11]. Since TMM and the ERECTA family are found in *P. patens*, in which stomata are distributed around the sporophyte [12], it is strongly deduced that the ligand-receptor module was developed in early land plants. It is interesting that the dramatic increase in stomatal density that occurred in early vascular plants in the late Devonian [3] appears to coincide with the evolutionary acquisition of AtEPFL9/Stomagen-like peptide, which positively regulates stomatal development. Beerling et al. [4] hypothesize that the increased stomatal density on leaf surfaces was an essential driving force for evolving megaphyll leaves in vascular plants. The acquisition of an *AtEPFL9/Stomagen*-like gene would be potentially advantageous for this morphological innovation because the peptide hormone Stomagen is a potent inducer of leaf stomata [10]. Our findings provide new insights into functional divergence in the molecular evolution of the *EPF/EPFL* family genes and will facilitate future studies on the evolutionary development of stomata in land plants.

## Supporting Information

Figure S1 **Phylogenetic tree of** ***EPF/EPFL*** **genes.** The phylogenetic tree was reconstructed by the NJ method from the numbers of amino acid substitutions estimated by the JTT model. The numerals at the branch indicate bootstrap values calculated by the NJ method with 1,000 replications. Bootstrap values >50% are shown.
(EPS)

Figure S2 **Molecular properties of** ***P. glauca*** **EPF2.** (A) Ribbon model and (B) MD simulation of PgEPF2.
(EPS)

Table S1 ***EPF/EPFL*** **genes used in the phylogenetic analyses.**
(XLSX)

Table S2 **Putative signal peptide and cleavage site of the proteins encoded by genes in the** ***EPF1-EPF2-EPFL7*** **clade and the** ***EPFL9/Stomagen*** **clade as predicted by the SignalP 4.0.**
(XLSX)

Dataset S1 **Predicted** ***EPF/EPFL*** **genes in land plants.** *EPF/EPFL* genes were retrieved from genomic databases for *Arabidopsis thaliana* (The Arabidopsis Information Resource, TAIR), *Oryza sativa* (Rice Annotation Project Database), *Picea glauca* (The Gene Index Project) and *Carica papaya*, *Medicago truncatula*, *Populus trichocarpa*, *Physcomitrella patens*, *Sorghum bicolor* and *Selaginella moellendorffii* (Phytozome v8.0).
(TXT)
